# Substituent Effect on Porphyrin Film-Gas Interaction by Optical Waveguide: Spectrum Analysis and Molecular Dynamic Simulation

**DOI:** 10.3390/ma13245613

**Published:** 2020-12-09

**Authors:** Nuerguli Kari, Marco Zannotti, Gulgina Mamtmin, Rita Giovannetti, Babak Minofar, David Řeha, Patigu Maimaiti, Buayishamu Kutilike, Abliz Yimit

**Affiliations:** 1 Institute of Applied Chemistry, College of Chemistry, Xinjiang University, Urumqi 830046, China; nurri7695@163.com (N.K.); gulgina125@sina.com (G.M.); 18195918820@stu.xju.edu.cn (P.M.); ayisha29@sina.com (B.K.); 2 Chemistry Division, School of Science and Technology, University of Camerino, 62032 Camerino, Italy; 3 College of Chemistry and Environmental Science, Kashgar University, Kashgar 844006, China; 4 Center for Nanobiology and Structural Biology, Institute of Microbiology, Academy of Sciences of the Czech Republic, Zamek 136, 37333 NovéHrady, South Bohemia, Czech Republic; reha@nh.cas.cz

**Keywords:** substituent effect, meso-phenyl porphyrins, optical waveguide, gas sensor, molecular dynamics simulation

## Abstract

Substituent effect on optical gas sensing performance in porphyrin-based optical waveguide detection system was studied by molecular dynamics simulation (MDS), absorption/emission spectrum analysis, and optical waveguide (OWG) detection. The affinities of porphyrin with seven types of substituents (–H, –OH, –tBu, –COOH, –NH_2_, –OCH_3_, –SO_3_^−^) on para position of meso-phenyl porphyrin toward gas molecules in adsorption process were studied in different size of boxes with the same pressure and concentration. Analyte gases (CO_2_, H_2_S, HCl, NO_2_) were exposed to porphyrin film in absorption spectrophotometer, and in OWG with evanescent field excited by a guiding laser light with 670 nm wavelength. The extent of interaction between host molecule and the guest analytes was analyzed by the number of gas molecules in vicinity of 0.3 nm around substituents of porphyrin molecules. Optical waveguide results reveal that sulfonate porphyrin is mostly responsive to hydrochloride, hydrosulfide gas and nitrogen dioxide gases with strong response intensity. Molecular dynamics and spectral analysis provide objective information about the molecular state and sensing properties. Molecular rearrangements induced by gas exposure was studied by spectral analysis and surface morphology before and after gas exposure taking hydrosulfide gas as an example. Film-gas interaction mechanism was discussed in terms of each gas and substituent group characters.

## 1. Introduction

Optical waveguide sensor (OWGS) is useful in detecting foreign molecules based on sensing materials chemical-optical properties and evanescent field with guided light [1,2,3,4,5]. Being the main part of OWGS, sensing material has to be chosen carefully, considering refractive index [6], light transmittance characters [7], nonlinear optical properties [8] to the formation of guided light and transform the chemical signals to electric data. As a nonlinear optical limiter, porphyrins [9] are one of the proper choices for trapping laser light and guiding wave to form optical waveguide sensors. Porphyrin molecules are active in interacting with foreign molecules and ions [10], especially with those which are small in size to fit the inner space of porphyrin core, and with those that are able to form hydrogen bonds or interacts through electrostatic forces [11,12,13,14,15]. All of these features allow researchers to mimic porphyrin related biological processes such as photochemical processes [16,17], adsorb gaseous compounds [18] to serve as gas storage. Besides, porphyrins in film state exist in aggregated form, which could be tuned by simple ways such as varying solvents or concentration [19,20,21]. Another way to transform or exaggerate number of active sites is functionalization of the sensing material.

Several dyes and pH indicators were studied to detect organic volatiles and inorganic vapors using optical waveguide system [16,17,19,20,21,22,23,24,25,26,27]. Herein, influence of substituents on porphyrin molecules to the sensitivity and selectivity has been analyzed with molecular dynamic simulation (MDS). As the sensing part in OWGS, porphyrins are active to form covalent and non-covalent interactions with small molecules, performing quite good sensitivity and selectivity, with fast response and recovery. State of porphyrins as thin film on optical waveguide substrate influences the sensitivity toward analytes, since accessible active sites on porphyrin or the porosity are dependent on the number of free molecules and type of aggregation. These factors determine the light absorption ability and refractive index of porphyrin film, affecting the evanescent wave intensity in guiding wave of OWGS that means intensity of response signal reflects the state of porphyrins in film [28,29].

Substitute types on porphyrin molecules, central metallic atoms, protonation, or deprotonation degrees are the main factors that depend on the main interactions and sensitivities [25]. State of porphyrin molecules is also affected by film forming methods [30], solvent types [31], and substituted groups [32]. As a result, these factors influence the guiding light mode, response intensity, and response-recovery time. Therefore, understanding the effect of these factors is helpful to optimize sensing properties. Intermolecular interactions between thin film sensing layer and the analyte molecules were studied by a quantitative structural property relationship (QSPR) approach in an integrated optical Bragg grating detector, which was based on the change of evanescent wave and refractive index [33,34]. Different substituted cyclodextrins serving as the affinity materials in optical Bragg solvent vapor sensor were studied with comparison of suitability in terms of response behavior [35,36,37,38]. Herein seven types of porphyrins molecules with seven different substituents on para-position of phenyl part, 5, 10, 15, 20–(tetra–4–R–phenyl) porphyrin respectively recorded as R=H (TPP), –OH (THPP), –tBu (TBPP), –COOH (TCPP), –NH_2_ (TAPP), –OCH_3_ (TMPP), –SO_3_^−^ (TSPP) are reported.

In OWGS, film-analyte interaction dynamics are recorded by following the evanescent transformation, which was excited by laser light with certain wavelength, which is generally chosen based on the highest absorption intensity divergence between pure film and gas-exposed-film. When sensing film exposed to analyte gases, several properties of the film such as the thickness, refractive index, molar absorption coefficient [39] suffer changes following with color transformation, leading to attenuation or enhance of the evanescent absorbance intensity within the film [25]. Herein, molecular dynamic simulations offer theoretical proof for the sensing behavior in OWGS, in terms of the number of analyte molecules adsorbed in the vicinity of porphyrin molecules. Film-gas interactions in OWGS is simulated regarding the film as molecular clusters adsorbing analyte gas molecules through non-covalent interactions such as hydrogen bonding, Van der Waals force, and electrostatic interactions.

To optimize and explain the sensitivity, selectivity, and response properties both from the point of substituent on variance in porphyrin sensing film and from the point of analyte gas characters, optical waveguide detection experiment results were analyzed, combining with spectroscopic analysis both in liquid state and film state. All these results were compared to reported gas detection results, to analyze possible factors that influence the selectivity and sensitivity together with substituent effects.

## 2. Materials and Methods

### 2.1. Reagents

**Figure 1:**
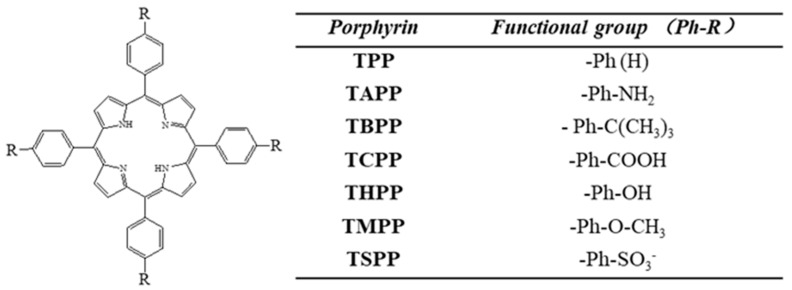
Chemical structure of investigated porphyrins. R=H (TPP), –OH (THPP), –tBu (TBPP), –COOH (TCPP), –NH_2_ (TAPP), –OCH_3_ (TMPP), –SO_3_^−^ (TSPP).

Meso–5, 10, 15, 20–(tetra–4–aminophenyl) porphyrin (TAPP, 98%) powder was purchased from Ji Lin Yan Shen Technology Co., Ltd., (Jilin, China). Meso–5, 10, 15, 20–(tetra–4–methoxyphenyl) porphyrin (TMPP, 95%) powder was provided by Aladdin Technology Co., Ltd. (Shanghai, China). Meso–5, 10, 15, 20–(tetra–4–carboxyphenyl) porphyrin (TCPP, 97%) was purchased from Shanghai Mairuier Chemical Technology Co., Ltd (Shanghai, China). Other Porphyrins meso–5, 10, 15, 20–(tetra–4–hydroxyphenyl) porphyrin (THPP, 95%), meso–5, 10, 15, 20–(tetra–4–t–butylphenyl) porphyrin (TBPP, 95%), meso–5, 10, 15 ,20–(tetra–4–sulfonato phenyl) porphyrin (TSPP, 95%), meso–5, 10, 15, 20–(tetra–phenyl) porphyrin (TPP, 97%) were purchased from Sigma-Aldrich Company, Ltd. (Shanghai, China). All the porphyrins used, and the relative functional groups are listed in Figure 1.

Analyte gases were prepared by collecting the gases in reactions at glass container (60 mL) with overdose use of CaCO_3_, FeS, Cu powders, smaller amount of HCl (12.4 mol/L) and nitric acid (14.4 mol/L). Then they were diluted to obtain the desired concentration, which was confirmed using commercial detection tubes (Gastec, Beijing Municipal Institute, Beijing, China). Hydrochloric acid (HCl) gas was prepared by vaporizing the concentrated solution (12.4 mol/L) naturally and diluted to the desired concentration. Gas preparation interactions are listed below:

(1) $$\begin{array}{l} {\mathrm{CaCO}}_{3(s)}+2{\mathrm{HCl}}_{\left(\mathrm{aq}\right)}\to {\mathrm{CaCl}}_{2}{}_{\left(s\right)}+H_{2}O_{\left(l\right)}+{\mathrm{CO}}_{2(g)}\\ {\mathrm{FeS}}_{\left(s\right)}+2{\mathrm{HCl}}_{\left(\mathrm{aq}\right)}\to {{\mathrm{FeCl}}_{2}}_{\left(s\right)}+H_{2}S_{\left(g\right)}\\ {\mathrm{Cu}}_{\left(s\right)}+4{\mathrm{HNO}}_{3}{}_{\left(\mathrm{aq}\right)}\to \mathrm{Cu}{\left({\mathrm{NO}}_{3}\right)}_{2\left(s\right)}+2H_{2}O_{\left(l\right)}+2{\mathrm{NO}}_{2}{}_{\left(g\right)} \end{array}$$

### 2.2. Molecular Dynamic Simulations

To study the adsorption of inorganic molecules in gas phase at the surface of sensing film which was porphyrin molecules with different substituents (Figure 1), classical molecular dynamics (MD) simulations have been performed.

All the MD simulations were performed in slab geometry with different size of boxes in order to guarantee that the systems have similar pressure as gas molecules have different sizes; therefore, the boxes have different dimensions in which the z direction of box was elongated to ensure the partial pressure of the system. In all prepared systems, only one porphyrin molecule with different substituents and 1000 to 3000 gas molecules was studied to ensure that all systems have same concentration of gases.

As the MD simulations have been performed for gas adsorption, all porphyrin molecules except the porphyrin with sulfonate (SO_3_^−^) substituents were assumed to be neutral, therefore no counter-ions were used to neutralize the system, while for neutralizing SO_3_^−^ substituents four sodium counter-ions were added to the system. Our objective is to calculate the affinity of porphyrin molecules toward different gas molecules using the number of gas molecules around different substituents of porphyrins determined from trajectories of MD simulations.

To quantify the interaction of gas molecules with porphyrin molecules to address the affinity for different gas molecules, the same parameter sets for all molecules for all MD simulations were used by applying the General Amber Force Field (GAFF) model [40], which shows good bulk and surface properties for different gas and liquid molecules [41,42,43,44] were used for all molecules throughout the all MD simulations.

To obtain partial charges for porphyrin, inorganic and organic molecules which are used in adsorption process, ab initio geometry optimization using the Gaussian 03 package [45] was performed, employing the B3LYP/cc–pVDZ method. Atomic chargers were calculated for optimized geometries by Restrained Electrostatic Potential (RESP) fitting scheme [46] using the Antechamber program [47] of the Amber program package (version 1.27). The porphyrin molecule was randomly put into the simulation box and gas molecules were added to the system to ensure the same molar concentration of all gas molecules in the systems. Packmol package was applied [48,49] in order to achieve the random distribution of porphyrin and gas molecules in the simulation box. First of all, steepest descent minimization procedure was used to avoid all unfavorable contacts and interactions in the solutions and all systems were minimized to proceed to equilibration which was 500 ps NPT (isothermal-isobaric ensemble) restrained simulations. After finishing the NPT simulation in order to have bulk solution the slab geometry of all systems was made by elongation of z diminution of the box. Linear constraint solver (LINCS) algorithm [50] was employed for all bonds involving hydrogen atoms. The short range non-bonded interactions were truncated to zero with the cutoff distance of 1.2 nm, and the long-range part of the electrostatic interactions was calculated by the particle mesh Ewald method [51]. Initial velocities were given to the molecules in all systems according to Maxwell–Boltzmann distribution at 300 K. V-rescale coupling algorithm was used [52] with the coupling constant of 0.1 ps to maintain constant temperature and pressure for all simulated systems. All production runs were performed in NVT ensemble (Canonical ensemble) for 50 ns at 300 K, and a time step of 2 fs was used for all simulations. Coordinates, velocities, and energies were saved for analysis every 5 ps. All simulations were performed employing Gromacs 4.6.5 program package [53,54,55]. Visual Molecular Dynamics (VMD, version 1.9.3) program was used for visualizations of the trajectory and preparation of snapshots [56].

### 2.3. Spectrum and Morphology Analysis

Double beam UV-Vis spectrophotometer (UV-1780, Shimadzu, Japan) was used at room temperature scanning range as 300 nm–800 nm, medium scanning speed, slit width 2 nm to analyze each porphyrin in solution state and their aggregation in film state before and after gas exposure. Selection of a proper solvent for each porphyrin was investigated considering effects of substituents on solubility of porphyrins, and film forming ability of solvents in spin-coating process (spin coater KW-4A, Shanghai Kaimeite Artificial China Technology Company, Shanghai, China). Dimethylformamide (DMF), tetrahydrofuran (THF), chloroform, dichloromethane and methanol were studied to select the proper solution for each porphyrin. Aggregation state of porphyrins deposited on potassium ion exchanged glass substrate was further studied by Scanning Electronic Microscope (SEM).

### 2.4. Optical Waveguide Detection

Sensing film of each porphyrin were achieved by deposition of their solution (1.3–1.7 × 10^−3^ mol L^−1^) separately on one of the surfaces of K^+^ ion exchanged (K^+^ layer depth 1~2 µm) soda-lime glass (76 × 26 × 1 mm) to exaggerate the refractive index of the substrate approximately 0.01. Sensing surface area is the same for all, as 2.6 × 1 mm^2^, and spin coater (KW-4A-type, Shanghai Chemat Technology Ltd., Shanghai, China) was used in vacuum with the first rotating speed as 500 rpm for 5 s, and second rotating speed as 1300 rpm for 25 s.

**Figure 2:**
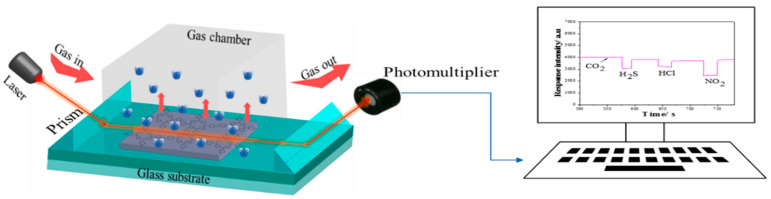
Schematic diagram of the homemade optical waveguide (OWG) detecting system.

Optical waveguide sensing experiment was performed on homemade detection system, Figure 2, which was composed of light of a semiconductor laser (670 nm, 10 mW, beam width as 1 mm) to transform and record the change within the sensing layer. For reducing the optical loss, a coupling material diiodomethane (n = 1.74) was used between the prisms (n = 1.79) and the substrate waveguide (n = 1.52) [1]. Gas chamber with 2 cm^3^ volume was used as an interaction room; gas injection was controlled with a flowmeter connected to the gas chamber at one end and the other to a plastic syringe with 20 cm^3^ volume, which was replaced with a new one for each gas to avoid cross-influence. After each gas exposure, ammonia gas (saturated) was injected to recover the film to the initial state according the typical bands in absorption spectrum [25]. Dry air was used as carrier gas and as diluter in the flowing system. All refractive indexes provided in this work are for the wavelength 589.3 nm (mean of sodium D lines) at a pressure of 101.325 Pa and 298K, Table 1 [57,58]. The circumstance around the film before each gas exposure was kept isothermal (298 K) and dry using air conditioner equipment and rinsing with standard dry air in the gas chamber.

Film absorbance and morphology before and after gas exposure were examined using spectrophotometer (UV-1780, Shimadzu, Kyoto, Japan) and field emission Scanning electron microscope (FE-SEM; SU8019, Hitachi, Tokyo, Japan). Optical waveguide response was detected at room temperature and at dry atmosphere on the self-assembled gas detection system.

## 3. Results and Discussion

### 3.1. Molecular Dynamics Simulation Analysis

**Figure 3:**
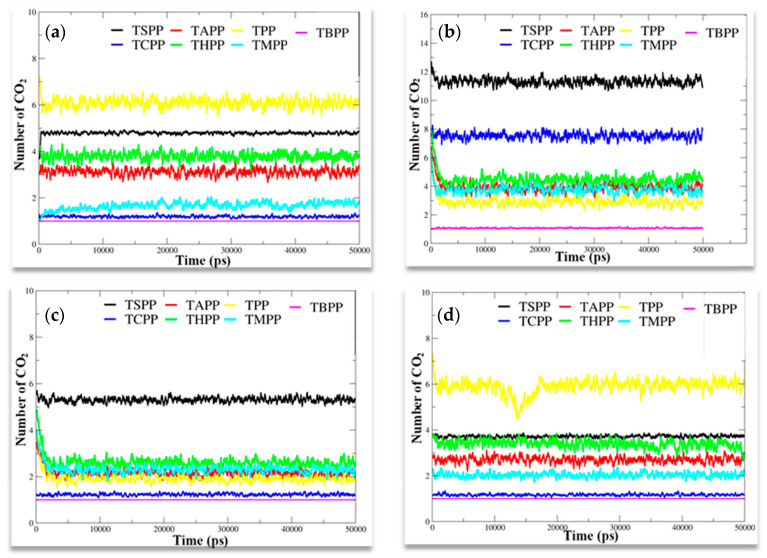
Number of (**a**) CO_2_, (**b**) H_2_S, (**c**) HCl, (**d**) NO_2_ gas molecules in vicinity of 0.3 nm around substituents of porphyrin molecules with different substituents.

Substituent effect was analyzed by MD simulation, which was optimized by the calculation of gas molecule numbers near the substituents on porphyrin macrocycle. The number of gas molecules, either inorganic or organic, around the substituents of different porphyrins can show the affinity of molecule to particular substituent, therefore the number of molecules in the vicinity of substituent can describe the extent of interaction. The Figure 3 shows the number of gas molecules in vicinity of 0.3 nm around the substituents of different molecules during the simulation time.

From Figure 3a it can be concluded that CO_2_ molecule is the most adsorbed at the surface of porphyrin molecule with H substituent (TPP), followed by porphyrin with SO_3_^−^ group (TSPP) then by porphyrin with OH (THPP) and NH_2_ (TAPP) groups while TCPP, TMPP and TBPP porphyrin have the lowest affinity. In the case of H_2_S (Figure 3b), the TSPP porphyrin with SO_3_^−^ group shows the strongest affinity; therefore, the number of gas molecules around SO_3_^−^ substituents is the biggest, followed by porphyrin with COOH group (TCPP) while THPP, TAPP, TMPP and TPP show similar affinity, TBPP has the least affinity toward H_2_S. From MD analyzed data, HCl gas adsorbs better at the surface of TSPP, followed by THPP, TMPP, TAPP, TPP that show similar affinity, and TCPP and TBPP have the lowest affinity (Figure 3c). The NO_2_ has the strongest affinity with TPP, followed by TSPP and THPP with similar affinity; lower affinity was showed by TAPP and TMPP, TBPP and TCPP showed very low affinity for NO_2_ gas molecules (Figure 3d). All the analytes under study show lower or not affinity for TBPP. From Figure 3, it can be concluded that tertiary butyl functionalized porphyrin molecule has the least affinity for all the inorganic molecules.

In summary, MD simulation results in Figure 3 show that t–butyl substituted porphyrin (TBPP) has no affinity to any of the analytes that can be recorded as one molecule around a porphyrin molecule; The CO_2_, NO_2_ and functional groups with electron withdrawing groups such as –H, –OH, –NH_2_, –SO_3_ display strong affinity, others with electron donating groups show less affinity; –SO_3_ and –COOH porphyrins displays strong affinity to H_2_S, whereas, toward HCl porphyrins with SO_3_^−^ it is mostly attractive.

### 3.2. Spectrum Analysis

Considering hydrophilic and hydrophobic properties of investigated substituents on porphyrin marcocyles, we experimented on all porphyrins with each solvent by dissolving (some are even not soluble even with sonication), together with spectroscopic spectrum to observe the state of porphyrins in solvents and fabricating films by vacuum spin-coating with the dissolved solvent. We found that porphyrins with hydrophobic properties such as TPP, TBPP, TMPP dissolve well in low polar solvents such as dichloromethane (polarity index, PI = 3.1); and those with hydrophilic groups such as TCPP, THPP shows well solubility and film formation with methanol (PI = 5.1); TAPP and TSPP powders dissolve well and is able to form uniform film by spin-coating with higher polar solvents such as THF (PI = 4.0) and DMF (PI = 6.4) respectively. An optimal solvent was selected for the film fabrication: dichloromethane for TPP, TBPP and TMPP porphyrins, THF for TAPP porphyrin, methanol for TCPP and THPP porphyrins and DMF for TSPP. To have information about the spectral changes after the film preparation, all the porphyrins were spectrophotometrically monitored. As reported in Figure 5, all the porphyrins in solution (red line) show an intense Soret band in the range 418–431 nm and the characteristic four Q bands in the region of 500–700 nm. For each porphyrin molar absorption coefficient related to Soret and Q bands (ε) was calculated by linear calibration in the specific solvent of preparation (Figure S1); all the results are reported in Table 2.

**Figure 4:**
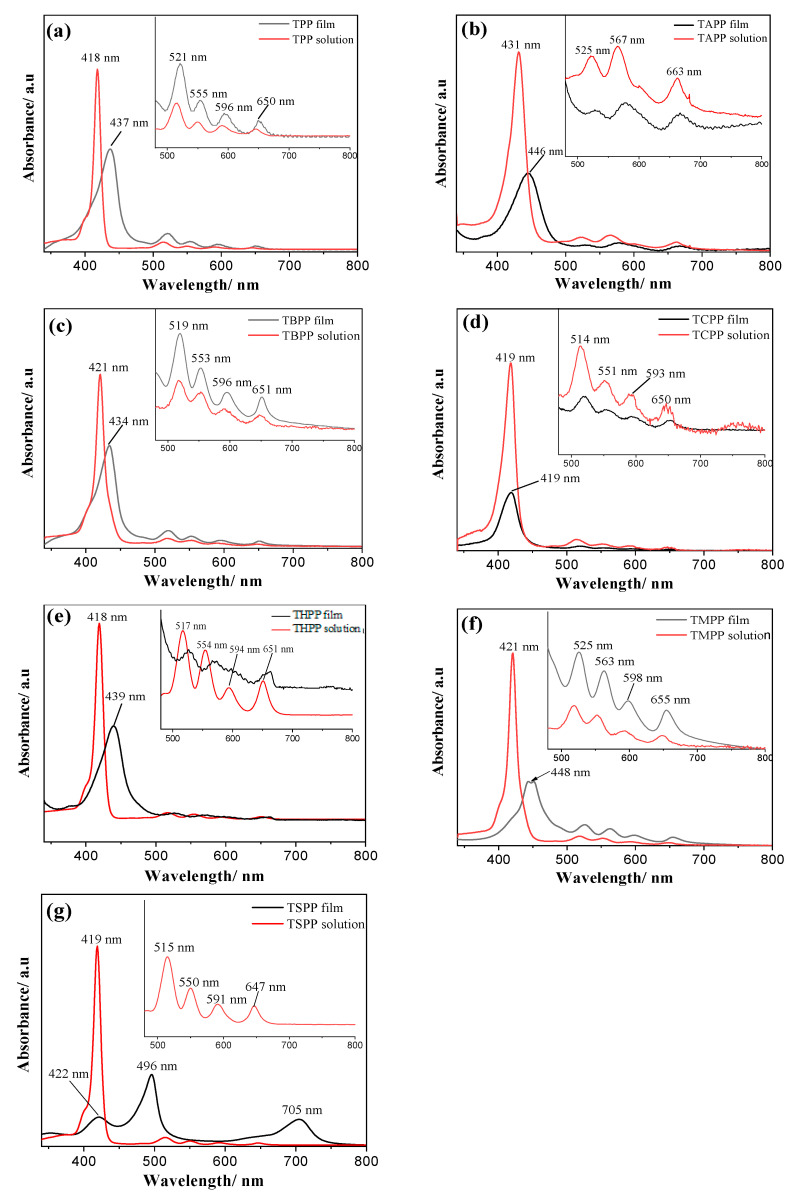
Absorbance spectrum of each porphyrin in film state and in solution with optimal solvents: (**a**) TPP in CH_2_Cl_2_, (**b**) TAPP in THF, (**c**) TBPP in CH_2_Cl_2_, (**d**) TCPP in CH_3_OH, (**e**) THPP in CH_3_OH, (**f**) TMPP in CH_2_Cl_2_, (**g**) TSPP in DMF.

As can be seen in Figure 4, after deposition of porphyrins by spin-coating on the substrate, spectral changes can be observed in the Soret band of all porphyrins that are red shifted and broadened compared to their solution states, except for TCPP film which stay the same maximum wavelength as in the liquid form. In terms of Q bands, shape and position remain the same for TPP, TBPP, TCPP and TMPP, small changes have been observed for TAPP and THPP, while major changes are present in TSPP film where the four Q bands replaced with only one strong band at 705 nm. The red shifts of Soret band are peculiar of the formation of J-aggregates that are edge-by-edge or side-to-side assemblies producing bathochromic shift [59]. The results obtained for TSPP film are in accordance with the formation of J-aggregates as reported in Arai et al. [60]. 

**Figure 5:**
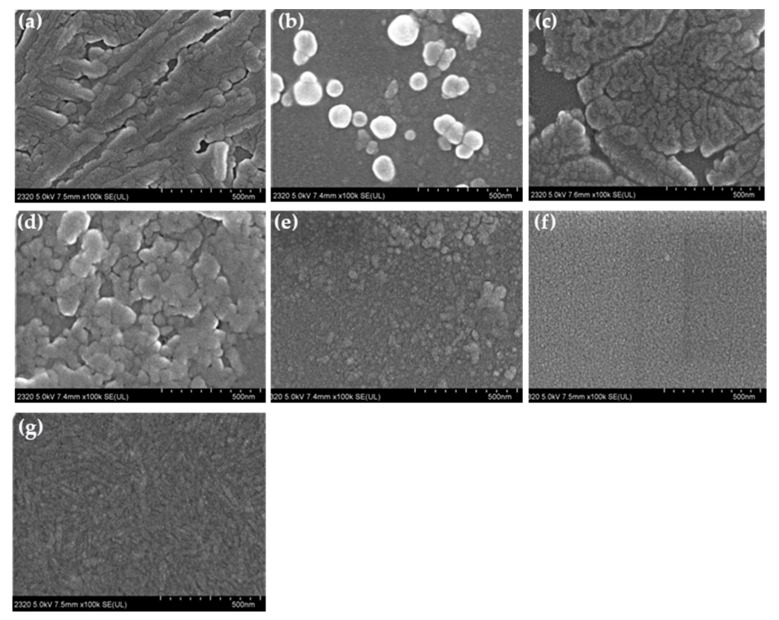
SEM morphology of each porphyrin in film state (**a**) TPP, (**b**) TAPP, (**c**) TBPP, (**d**) TCPP, (**e**) THPP, (**f**) TMPP, (**g**) TSPP deposited on glass substrate by spin-coating (1600 rpm).

The influence of H, NH_2_, t–butyl, OH, and OCH_3_ substituents on the aggregation extent can be seen also as visualized by the morphological analysis of SEM images reported in Figure 5. As it is possible to observe from the figure, all porphyrins aggregate in a compact form on the substrate with different morphologies. TPP (Figure 5a) forms large and tree shape aggregates, TAPP (Figure 5b) shows not uniform particle aggregates with the presence of larger globular shape, TBPP (Figure 5c) forms large and undefined aggregates with large space, while TCPP (Figure 5d) is composed of small particles of about 50 nm. In addition, regular distribution of small particles is detected on THPP film (Figure 5e) with the presence of larger ones, TMPP (Figure 5f) shows uniform distribution of very small nanoparticles while regular surface composed of stratified rods are present in TSPP film (Figure 5g).

### 3.3. Gas Exposure

**Figure 6:**
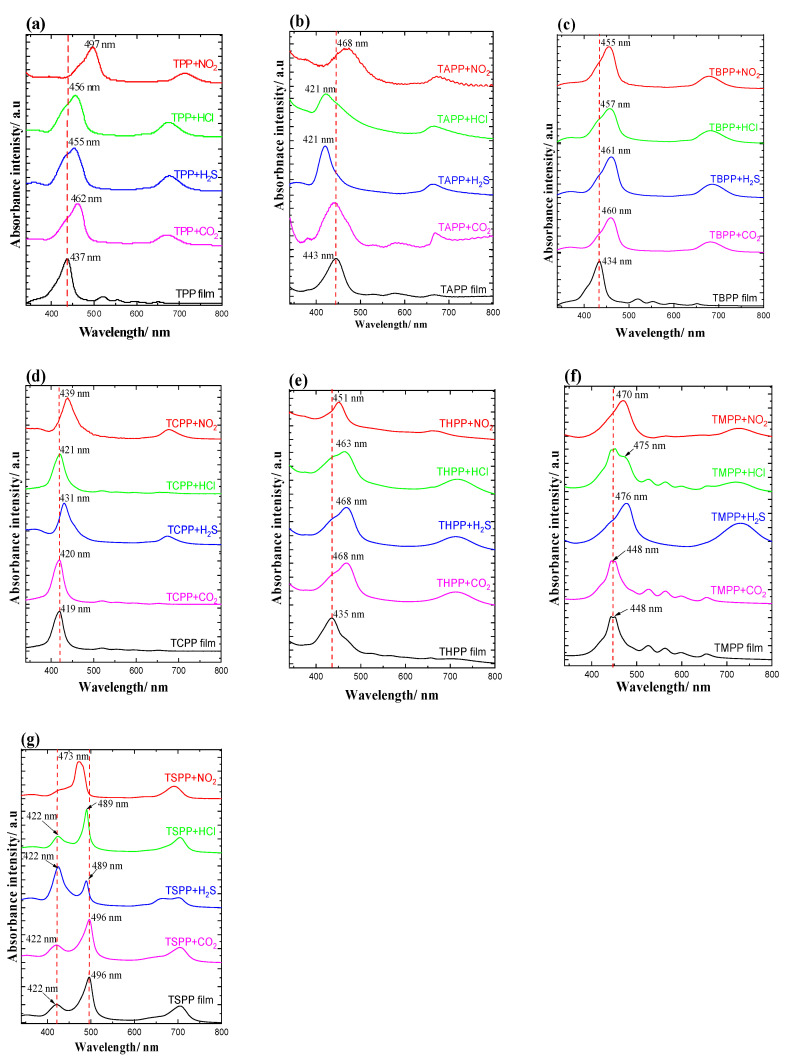
UV-Vis spectrometric response of porphyrin films exposing to analyte gases (100 ppm) (**a**) TPP, (**b**) TAPP, (**c**) TBPP, (**d**) TCPP, (**e**) THPP, (**f**) TMPP, (**g**) TSPP.

Absorbance changes before and after gas exposure of porphyrin films are reported in Table 3 and in the UV-Vis spectra in Figure 6, within 340–800 nm.

As is reported in Table 3 and Figure 6a, the Soret band of TPP film was red shifted when exposed to CO_2_, H_2_S, HCl, NO_2_ of 25, 18, 19 and 60 nm, respectively.

For TAPP film (Figure 6b), no remarkable change in position of bands with CO_2_ exposure; only a broadening of the Soret band was detected, which might be the result of the enlargement of the aggregated form of the porphyrins in film [24]. When TAPP was exposed to H_2_S and HCl, both Soret bands were blue shifted of 22 nm, while with NO_2_ it broadened with bathochromic shift of 25 nm. H_2_S, HCl, NO_2_ exposures decreased or replaced the Q bands between 500 nm and 600 nm, with rather strong band around 670 nm.

All gas exposure results of TBPP (Figure 6c) are remarkably similar with TPP film; bathochromic shift extent with CO_2_, H_2_S, HCl, NO_2_ is 25, 27, 27 and 21 nm respectively (Table 2).

In terms of TCPP (Figure 6d) porphyrin film, NO_2_ and H_2_S exposures resulted in 20 and 12 nm bathochromic shift of the Soret band, from 419 nm to 431 nm and 439 nm respectively, following with degeneration of Q bands and formation of a new band at 670 nm while HCl and CO_2_ caused no remarkable changes.

THPP film (Figure 6e) gas exposure results in the exhibition of similar behavior as TPP and TBPP, with red shift of 33, 33, 28, 26 nm for CO_2_, H_2_S, HCl and NO_2_, respectively. TMPP film (Figure 6f) shows similar behavior to TCPP film, with 26 and 18 nm bathochromic shift with H_2_S and NO_2_ exposure; only 1 nm change both for CO_2_, without any change in the shape of spectrum lines; in terms of HCl, there appears a shoulder at 475 nm at the right position of Soret peak at 448 nm, forming a peak at 670 with 0.007 absorbance intensity, while other Q bands remains without remarkable attenuation, as can be seen in Figure 6f.

TSPP film (Figure 6g) with H_2_S, HCl, NO_2_ shows a bathochromic shift of Soret band at 496 nm of 7, 6 and 23 nm, respectively. Also changes in the absorbance ratio of the two Soret bands with respect to TSPP film before exposure are observed as well, demonstrating that the exposure of gases modified the aggregate. In particular, the band at 496 decrease for various extent in intensity with all gas exposures, also the band at 422 nm decreases with CO_2_ and NO_2_ exposure, while that increase with HCl and H_2_S; in the presence of HCl, the ratio of peak intensity at 489 nm and 422 nm is 2.62 (ΔA_496_ = −0.10, ΔA_422_ = 0.09), while the band at 422 nm increases; on the other hand, the opposite behavior was detected with H_2_S exposure, (ΔA_496_ = −0.34, ΔA_422_ = 0.25) while the band at 473 nm is predominant in the presence of NO_2_.

**Figure 7:**
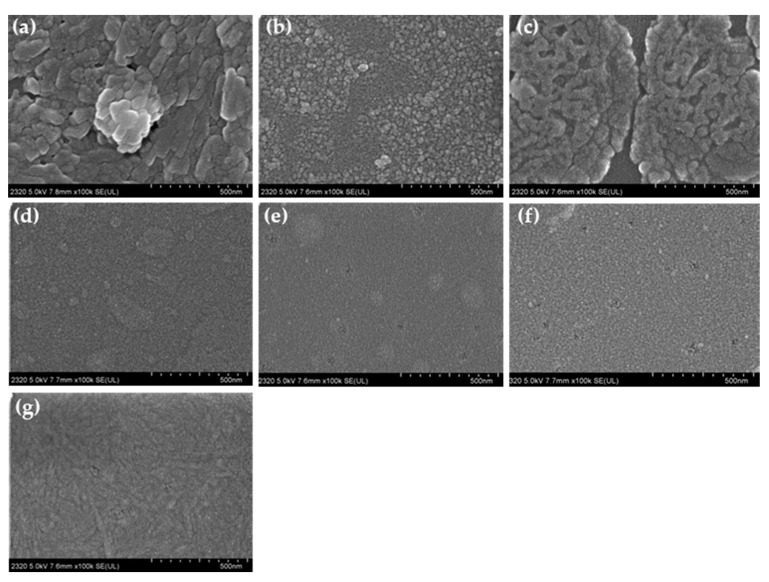
Morphology of each porphyrin film on potassium ion exchanged glass substrate after hydrogen sulfide (1000 ppm) gas exposure, (**a**) TPP, (**b**) TAPP, (**c**) TBPP, (**d**) TCPP, (**e**) THPP, (**f**) TMPP, (**g**) TSPP.

Morphology of the sensing film after H_2_S gas exposure, Figure 7, cluster size and space between clusters within film is distorted by gas molecules, causing thickening of film and transformation of refractive index. TPP porphyrin (Figure 7a) molecules grow vertically after interacting with H_2_S, which protonates free-base molecules and leads to new type of self-assembly of the protonated porphyrin molecules in dichloromethane solution-based film. In terms of –NH_2_ substituted porphyrin (TAPP) film (Figure 7b), when exposed to H_2_S gas, the aggregated balls deteriorate, forming more uniform particle distribution, indicating the interaction of surface with the guest molecules; TBPP film surface (Figure 7c) presents more holes as corroded with H_2_S exposure, which protonate porphyrins molecules making previous aggregates incompact; COOH substituted (TCPP) film, (Figure 7d) surface was collapsed to smaller globes with H_2_S exposure; OH substituted porphyrin (THPP) (Figure 7e), spheres were as well collapsed into much smaller balls with H_2_S gas adsorption; on methoxy substituted (TMPP) film surface (Figure 7f), there appears dozens of holes as if eroded by H_2_S gas; TSPP (Figure 7g) molecule aggregates grow longer, up to interacting with H_2_S. These transformations in film morphology with gas exposures explain the behavior of film-gas performance in absorption spectrum discussed above and the response characters in OWGS. As morphology changes with gas exposure, light absorbing and scattering ability varies in comparison of the one without gas interaction, resulting in change of evanescent wave and output light intensity in OWGS.

**Figure 8:**
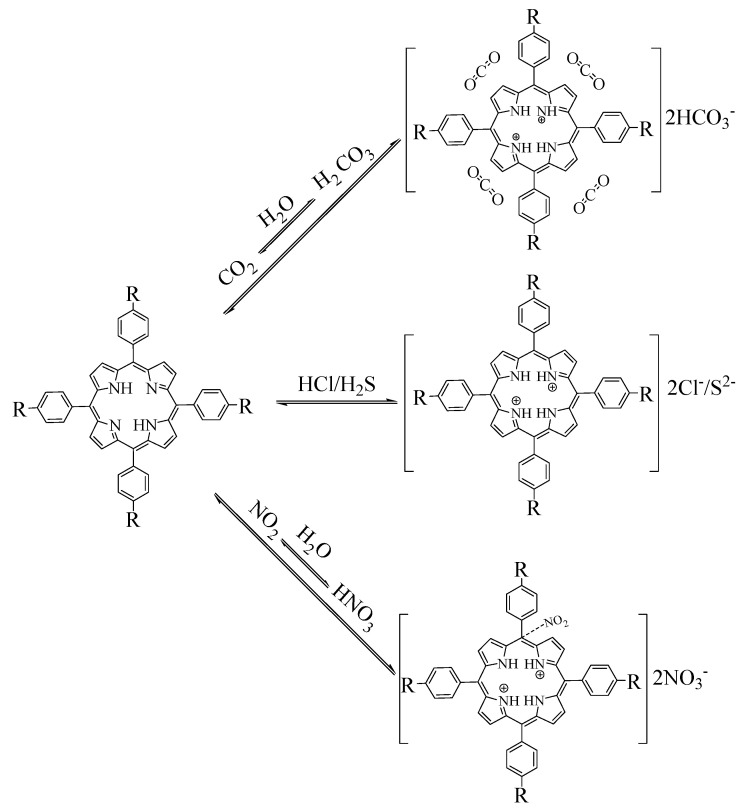
Interaction between porphyrins and analyte gases.

Film-gas interaction probably due to protonation of porphyrin film by hydrochloric and hydrogen sulfide, oxidation, protonation and electron transference with nitrogen dioxide and carbon dioxide molecules, is shown in Figure 8. Interaction mechanism between metal-free porphyrin and carbon dioxide is probably due to the rather weak π-π interaction. Thanks to the adsorption of these gas molecules, energy gap (ΔE) between S_0_ and S_2_ might be attenuated. In addition, another obvious phenomenon in this series is the formation of a new peak at around 700 nm, with the elimination of all other Q bands between 500 nm and 650 nm. These are the characteristics of protonation of porphyrins [61], while in terms of CO_2_, NO_2_ gas exposures and porphyrin films exhibit typical protonation peaks, with all Soret bands red-shifted, especially strong for NO_2_ exposure (minimum 16 nm, as shown in THPP porphyrin). Therefore, protonation was regarded as the main cause due to the formation of carbonate acid and nitric acid thanks to the water molecules that were produced during gas preparation, as can be seen in Section 2.1. Researchers reported that the NO_2_ molecule might bind to one of the carbon atoms on meso position on porphyrin rings modifying the electron distributions on porphyrin macrocycle [62,63]. According to the spectral behavior of the film with gas exposures in our research, as can be seen in Figure 6, we regard the presence of protonation and formation of supramolecules that was composed of host and guest molecules [64]. Formation of the peculiar peak at 650–700 nm, especially strong for TPP and TBPP porphyrins, might be accused to the richer electron densities on these two macrocycles that was caused by the electron-pushing group such as t–butyl [65]. Besides, protonating ability of nitric acid (pk_a_ = −1.64) is stronger than that of carbonate (pk_a1_ = 6.35) [66], resulting in a strong response in NO_2_ exposure and rather weak change in CO_2_ exposure.

### 3.4. OWG Response Analysis

In OWG detection, a laser light with 670 nm wavelength was selected based on changes of absorbance intensity (ΔA) when exposed the film to analyte gases on UV-vis spectrum. Table 4 reports the results of absorbance change at 670 nm of the different porphyrin films after gas exposures in film-gas absorption spectrum.

**Figure 9:**
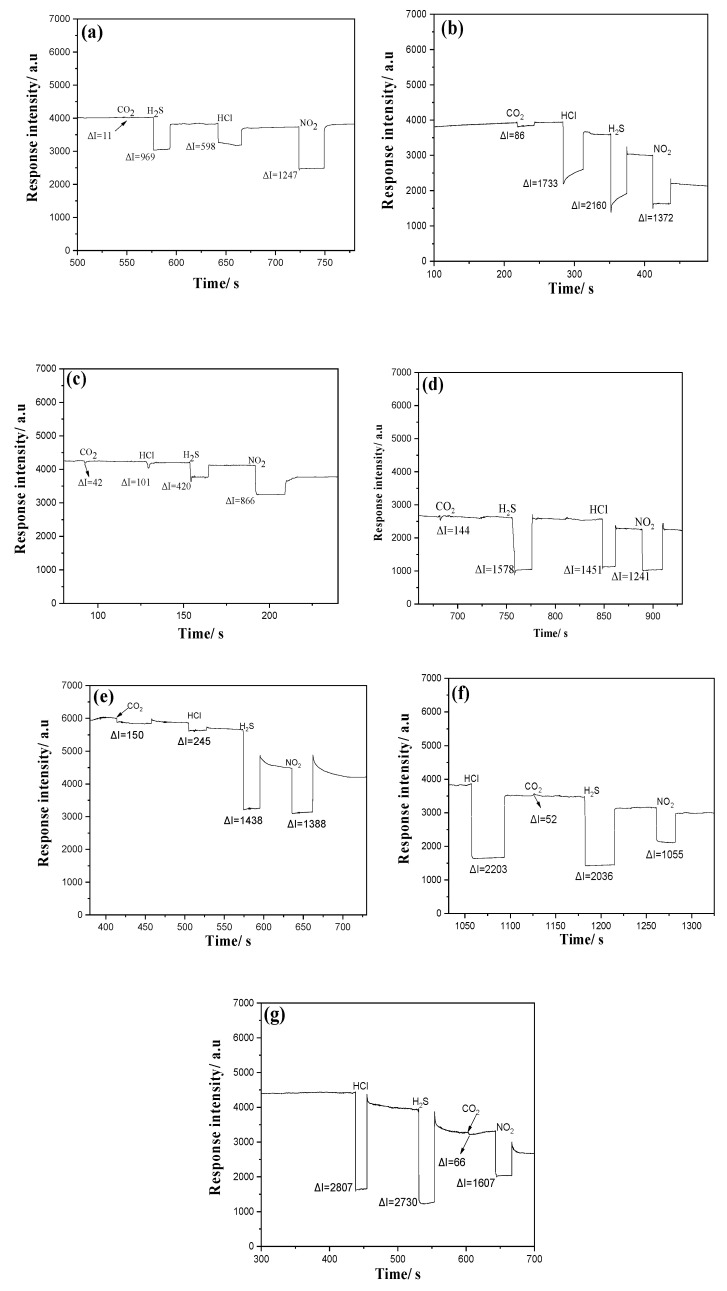
Response curve in optical waveguide (670 nm) with porphyrin film exposed to acidic gases (**a**) TPP, (**b**) TAPP, (**c**) TBPP, (**d**)TCPP, (**e**) THPP, (**f**) TMPP, (**g**) TSPP film.

As shown in Figure 9, output light intensity decreases in OWG when all these analyte gases interacted with film that was in coincident with absorbance intensity change in UV–vis spectrum exposing films to gases in a sealed cuvette. Considering the absorbance change, stability and availability of semiconductor laser lights, red light with wavelength 670 nm and 2 mm beam width at working distance was applied as light sources for all porphyrin films in gas detecting process. Adsorption process of gas molecules could be formulated based on a simple reaction–diffusion equation assuming a first order reaction of target gas [67], taking into account diffusion coefficient, rate constant, and film thickness, depth from the film surface, time, and gas concentration. OWG detecting system pictures dynamic curve describing sensitivity, which was expressed as output light intensity as vertical axis, and time factor as horizontal axis. The system was designed to form a guiding wave with zeroth mode within the thin film (thickness as 70–80 nm) when the propagation angle into the input prism is adjusted to the critical angle due to internal reflection, owing to the higher refractive indexes of porphyrins (>1.61, as listed in Table 1) than the substrate layer (n = 1.52) and that of the cladding air/gas layer (n = 1.00). The guided light propagating along the film encounters partially (with the height as a wavelength of the laser light 670 nm) into the cladding air, where an evanescent wave is formed, and the evanescent wave is strongly sensitive to surface conditions; these can be altered by exposing the film to different analyte molecules leading to remarkably different optical changes compared with the pre-exposed state. When electro-magnetic fields are separated with the critical angle, the zeroth mode of the output light shape on the photomultiplier screen reveals as a cross shape, the central light of which was recorded as the output light. The strength of the output light informs the response behavior of the deposited thin film interacting with the analyte molecules. This is because, the pre-exposed film absorbs less light at 670 nm wavelength, and gas-exposed film absorbs more, and less light reaches to the photomultiplier. Optical change of the film surface with gas exposure was exaggerated by evanescent filed, thus slightly optical changes can exhibit rather strong signals with the help of multi-photometer.

As given in Figure 9a, TPP film exhibits the strongest response toward NO_2_ (ΔI = 1247 a.u), then to H_2_S (ΔI = 969 a.u) and HCl (ΔI = 598 a.u), with only small response toward CO_2_ (ΔI = 11 a.u). TAPP film (Figure 9b) exhibits the weakest response to CO_2_ (ΔI = 86 a.u) as well, and the strongest to H_2_S (ΔI = 2160 a.u). From Figure 9c it is possible to observe that TBPP film displays the strongest response to NO_2_ (ΔI = 866 a.u) then to H_2_S (ΔI = 420 a.u), with quick recoverable weak signal to HCl (ΔI = 101 a.u) and CO_2_ (ΔI = 42 a.u). TCPP film (Figure 9d) shows small response to CO_2_ (ΔI = 144 a.u) as well, with strong responses to H_2_S (ΔI = 1578 a.u), HCl (ΔI = 1451 a.u) and NO_2_ (ΔI = 1241 a.u). Figure 9e shows the response behavior of THPP film that gives rather weak response to CO_2_ (ΔI = 150 a.u) and HCl (ΔI = 245 a.u) exposures, but strong to H_2_S (ΔI = 1438 a.u) and NO_2_ (ΔI = 1388 a.u). Figure 9f displays the response behavior of TMPP film that responds strongly to HCl (ΔI = 2203 a.u) and H_2_S (ΔI = 2036 a.u), then to as half as the former two gases toward NO_2_ exposure (ΔI = 1055 a.u), with as 40 magnitude smaller response to CO_2_ (ΔI = 52 a.u). In Figure 9g, TSPP film exhibits the strongest response toward HCl (ΔI = 2807 a.u) and H_2_S (ΔI = 2730 a.u) with slightly baseline shift after purging step, and the response is as strong as ΔI = 1607 a.u with NO_2_ exposure and as weak as ΔI = 66 a.u with CO_2_ exposure.

It can be observed from the response behavior (Figure 9) that NO_2_ gas exposures lead to remarkable baseline shifts with total recovery to the initial state in all films being unable to recover to the starting state but a new state near to the initial one with rinsing, while CO_2_ gas causes only a small response and total recovery in all porphyrin films. For this reason, the NO_2_ exposure is performed as the last one in the order, and CO_2_ as the first one in Figure 9a–e; while in Figure 9f,g, CO_2_ gas exposure was put in the middle to prove the response behavior after the other gas exposures, giving the same response behavior as putting at the first order. The difficulty in recovering with NO_2_ exposures can be ascribed to the stability of nitric form being unable to be deprotonated totally with ammonia exposure and recover back the initial state, but instead forms a new state connecting with ammonia molecules. However, the CO_2_ exposed form is not too stable and can drive back totally to the initial state.

Moreover, the exposure of the acidic gases with high concentration (1000 ppm) may lead to transformation of the aggregate form [68] of porphyrin molecules on the surface of the film as can be seen as an example of H_2_S exposure in morphology by SEM analysis (Figure 7) but also in the UV-Vis spectral changes (Figure 6). Specifically, in the case of TAPP, THPP and TSPP the hard recovery of the protonated form to the starting state associated with a baseline shift after ammonia exposure and dry air purging can be observed for all the gases except CO_2_. In fact, the baseline is going down compared to the very initial state when recovering with ammonia then with dry air flowing after HCl, NO_2_ and H_2_S gas exposures. In the case of TAPP (Figure 9b), the changes of aggregation type on porphyrin films especially after H_2_S, HCl and NO_2_ exposition (Figure 6b), probably is the main cause of the difficult restoration of the initial phase after the purge with ammonia and air. For THPP (Figure 9e), the baseline is going down compared to the very initial state; this may be ascribed to the stability of the protonated form of this porphyrin (THPP), which is unable to recover to the free form but another state forming supramolecules with ammonia [19]. In terms of TSPP film, the baseline shifting is remarkable, this might be due to the ionic properties of TSPP porphyrin associated with a changes of aggregation type as reported in the UV–Vis spectra (Figure 6g).

**Figure 10:**
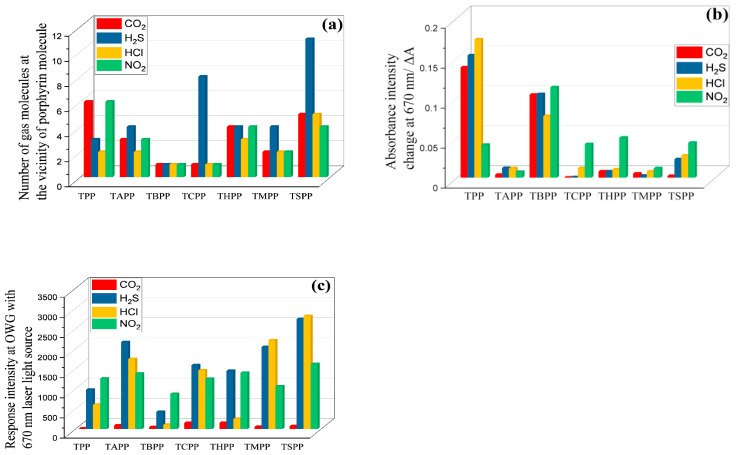
(**a**) Number of gas molecules in vicinity of 0.3 nm around each porphyrin molecule achieved by molecular dynamic simulation; (**b**) Absorbance change of porphyrin films after exposure to CO_2_, H_2_S, HCl, NO_2_ gases (saturated) at the 670 nm wavelength; (**c**) Response intensity change of each porphyrin films exposed to gases in optical waveguide with laser source as 670 nm.

As shown in Figure 10, in terms of NO_2_ exposures, TPP porphyrin displays the strongest affinity in MD simulation (Figure 10a), while TBPP porphyrin gives the highest absorbance change at 670 nm wavelength (the laser light source at OWGS), and TSPP porphyrin shows the strongest response. In terms of HCl and H_2_S interactions with porphyrin films, as given in Figure 10a, number of H_2_S and HCl gas molecules at the vicinity of TSPP porphyrin molecules are the highest than other gases and porphyrins that are in accordance with OWG response results in Figure 10c, though the absorbance change at 670 nm wavelength is not the highest among all porphyrins, as shown in Figure 10b. The deviation can be ascribed to the film surface morphology and optical absorbance or scattering abilities in UV–Vis and OWGS. The deviation between simulation and response results is in the case of CO_2_ gas exposure; in the simulation, TAPP, THPP, TSPP, and TPP molecules attract around three to six CO_2_ molecules around each porphyrin molecule that are higher than the other substituted porphyrins (Figure 3a). In OWG results all porphyrin films exhibit weak response to CO_2_ exposure (the highest is 150 a.u, Figure 10c). In this contest, refractive indexes [57] of these investigated gases are 1.00634 (H_2_S), 1.00447 (HCl), 1.000449 (CO_2_), 1.000297 (NO_2_), 1.00376 (NH_3_), 1.000292 (air) and 1.000256 (water vapor). The hypothesis that refractive index differences and the water vapor contents might be the reason for the deviation can be excluded. In addition, in the vicinity of 0.3 nm of porphyrin molecules the existing CO_2_ molecules are one to six (Figure 3a) depending on the type of substituent; however, in optical response the amount of adsorbed CO_2_ is too small for all porphyrin films. This behavior can be explained by the formation of new supramolecule between porphyrins and CO_2_ molecules that might possess very similar refractive indexes with the pre-exposed porphyrin films leading, therefore, small change in the output light and no remarkable response with CO_2_ exposure in OWG. This is also proved by the stability of the CO_2_ exposed films and the totally recovery of with ammonia rinsing.

In conclusion, OWG results summarized in Figure 10 show that TSPP film would be the optimal sensitive layer for H_2_S, HCl, and NO_2_ gases in optical waveguide with 670 nm laser light source at ambient temperature as confirm by MD simulation (Figure 10a). In addition, all porphyrins films with CO_2_ gas show very small response in OWG even with 1000 ppm concentration. The affinity achieved from MD for CO_2_ is instead strong with TPP and TSPP, large with TPP and TBPP porphyrin molecules. The MD results are in reasonable accord with spectral change (Figure 10) and, in this case, the low OWG intensity responses could be due to the fact that the wavelength at 670 is not optimal for the detection of CO_2_, or due to the formation of similar refractive indexes supramolecule porphyrins with CO_2_ molecules as the pre-exposed porphyrin films.

Researchers reported porphyrin films with various functional groups to detect H_2_S gas at room temperature with [22,23,24,25,26,27] using optical waveguide but the results and experimental performance are deficient in consistence. Therefore, in this work, we consolidate the experimental performing conditions including film width and thickness that was controlled by film fabrication velocity and solution concentration; laser light wavelength (670 nm), gas concentration was also unified to keep the only possible different variable is the influence of functional groups.

## 4. Conclusions

Substituent effect of porphyrin film on optical waveguide sensing system is investigated by molecular dynamic simulation analysis, molecular absorption spectrum analysis, and homemade optical waveguide detection system. Sulfonate porphyrin is optimal for hydrosulfide, hydrochloric gas, and nitrogen dioxide gas detection, while in terms of carbon dioxide gas. all porphyrins show quite a small response. The film–gas interaction mechanism considering aggregation of porphyrins molecules in film state, protonation by hydrochloric gas, hydrogen sulfide, carbonate acid and nitric acid in terms of CO_2_ and NO_2_ gas were discussed, supported by optical absorbance change and molecular dynamic simulations.

## Figures and Tables

**Table 1:** Refractive indexes (589.3 nm) of porphyrin and analyte gases (101,325 Pa, 298 K) [57,58].

Porphyrins	Refractive Index	Gas Analytes	Refractive Index
TPP	1.697	Air	1.000292
TAPP	1.762	H_2_S	1.000634
TBPP	1.610	HCl	1.000447
TCPP	1.744	NO_2_	1.000297
THPP	1.746	CO_2_	1.000449
TMPP	1.657	NH_3_	1.000376
TSPP	1.724	H_2_O (vapor)	1.000256

**Table 2:** Molar extinction coefficient (ε) results calculated at each band in corresponding solution with concentration range 7.65 × 10^−7^ ~ 6.12 × 10^−6^ mol L^−1^.

Porphyrins	Solvent	Molar Extinction Coefficient /ε (cm^−1^·mol^−1^·L)				
		Soret Band (1 × 10^5^)	Q_4_ (1 × 10^5^)	Q_3_ (1 × 10^5^)	Q_2_ (1 × 10^4^)	Q_1_ (1 × 10^4^)
TPP	CH_2_Cl_2_	417 nm 5.69	514 nm 1.95	548 nm 8.03	589 nm 5.32	645 nm 2.91
TAPP	THF	431 nm 1.87	523 nm 0.13	566 nm 0.97	-	661 nm 6.03
TBPP	CH_2_Cl_2_	421 nm 2.89	518 nm 1.16	553 nm 0.89	592 nm 4.34	650 nm 2.87
TCPP	CH_3_OH	416 nm 6.32	513 nm 3.52	548 nm 2.02	589 nm 1.42	645 nm 9.66
THPP	CH_3_OH	418 nm 6.16	517 nm 1.90	554 nm 1.49	594 nm 6.16	649 nm 7.91
TMPP	CH_2_Cl_2_	421 nm 2.90	518 nm 0.118	553 nm 0.90	592 nm 4.26	649 nm 2.87
TSPP	DMF	419 nm 7.19	515 nm 2.53	550 nm 1.35	591 nm 7.44	647 nm 6.84

**Table 3:** Maximum wavelength of each band of each porphyrin after gas exposure (10,000 ppm).

Porphyrins	Soret Band Before Gas Exposure (nm)	Soret Band After Gas Exposure (nm)			
	No Gas	CO_2_	H_2_S	HCl	NO_2_
TPP	437	462	455	456	497
TAPP	443	443	418	418	468
TBPP	434	460	461	457	455
TCPP	419	419	431	419	439
THPP	435	468	468	463	451
TMPP	448	448	476	475	470
TSPP	496	496	489	489	473

**Table 4:** Absorbance change at 670 nm of each porphyrin with gas exposures.

Film	Absorbance Change (ΔA) at 670 nm After Gas Exposure			
	CO_2_	H_2_S	HCl	NO_2_
TPP	0.1380	0.1530	0.1730	0.0410
TAPP	0.0036	0.0120	0.0116	0.0072
TBPP	0.1038	0.1045	0.0772	0.1130
TCPP	0.0001	0.0006	0.0119	0.0419
THPP	0.0078	0.0078	0.0104	0.0500
TMPP	0.0050	−0.0025	−0.0076	−0.0116
TSPP	−0.0021	0.0230	0.0277	−0.0436

## Supplementary Materials

The following are available on at https://www.mdpi.com/1996-1944/13/24/5613/s1, Figure S1: Absorption spectrum of porphyrin solutions with concentration range 7.65 × 10^−7^ ~ 6.12 × 10 ^−6^ mol·L^−1^ (a) TPP in CH_2_Cl_2_; (b) TAPP in THF; (c) TBPP in CH_2_Cl_2_; (d) TCPP in CH_3_OH; (e) THPP in CH_3_OH; (f) TMPP in CH_2_Cl_2_; (g) TSPP in DMF and the corresponding linear calibrations to calculation of molar absorption coefficients at each band equals to the slope of each linear equation.
