# Supplementary material for: Substituent Effect on Porphyrin Film-Gas Interaction by Optical Waveguide: Spectrum Analysis and Molecular Dynamic Simulation
Source: Materials (Basel). 2020 Dec 9;13(24):5613. doi: 10.3390/ma13245613 (PMC7763641; doi:10.3390/ma13245613)
Supplement: Supplementary file 1 [file materials-13-05613-s001.pdf]

materials

Substituent Effect on Porphyrin Film-Gas Interaction by Optical Waveguide: Spectrum Analysis and Molecular Dynamic Simulation

Nuerguli Kari [1], Marco Zannotti [2,*], Gulgina Mamtmin [1,3], Rita Giovannetti [2,*], Babak Minofar [4,*], David Řeha [4], Patigu Maimaiti [1], Buayishamu Kutilike [1] and Abliz Yimit [1,*]

[1] Institute of Applied Chemistry, College of Chemistry, Xinjiang University, Urumqi 830046, Xinjiang, China; nurri7695@163.com (N.K.); gulgina125@sina.com (G.M.); 18195918820@stu.xju.edu.cn (P.M.); ayisha29@sina.com (B.K.)

[2] Chemistry Division, School of Science and Technology, University of Camerino, 62032 Camerino, Italy; marco.zannotti@unicam.it

[3] College of Chemistry and Environmental Science, Kashgar University, Kashgar 844006, China

[4] Center for Nanobiology and Structural Biology, Institute of Microbiology, Academy of Sciences of the Czech Republic, Zamek 136, 37333 NoveHrady, South Bohemia, Czech Republic; minofar@nh.cas.cz

* Correspondence: ablizy@sina.com (A.Y.); marco.zannotti@unicam.it (M.Z.); rita.giovannetti@unicam.it (R.G.); minofar@nh.cas.cz (B.M.)

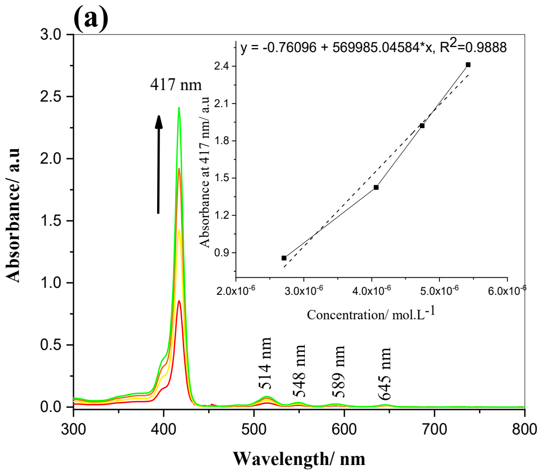

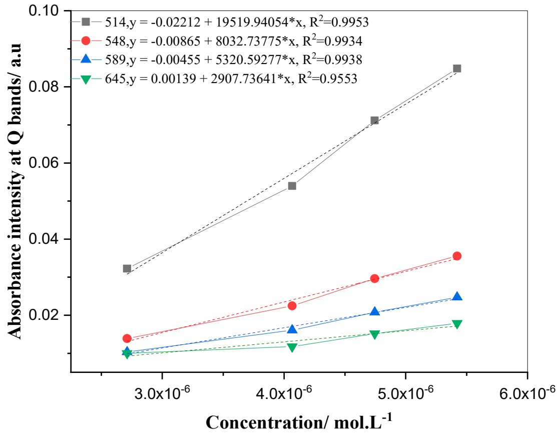

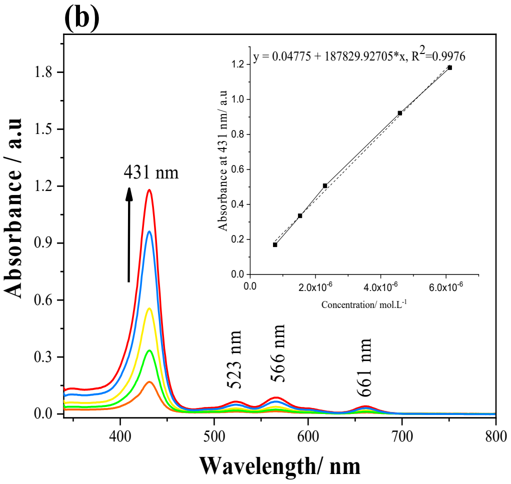
(b)
431 nm
523 nm
566 nm
661 nm
Absorbance / a.u
Wavelength/ nm
y = 0.04775 + 187829.92705*x, R2=0.9976
Absorbance at 431 nm/ a.u
Concentration/ mol.L-1

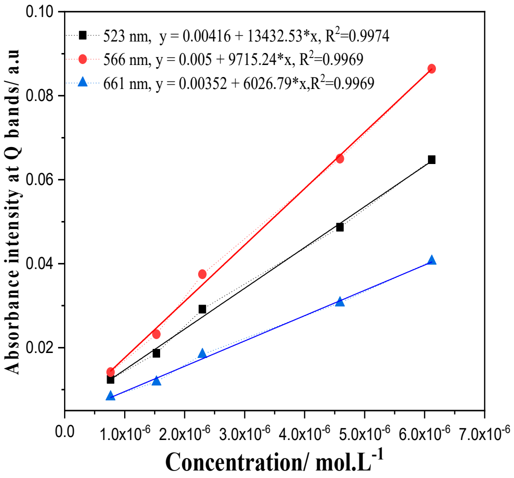
523 nm, y = 0.00416 + 13432.53*x, R2=0.9974
566 nm, y = 0.005 + 9715.24*x, R2=0.9969
661 nm, y = 0.00352 + 6026.79*x,R2=0.9969
Absorbance intensity at Q bands/ a.u
Concentration/ mol.L-1

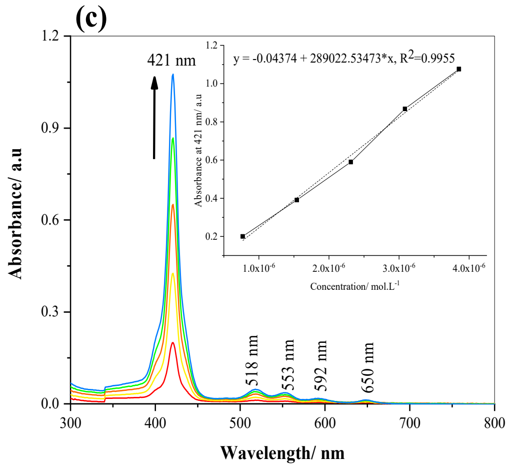
(c)
421 nm
518 nm
553 nm
592 nm
650 nm
Absorbance/ a.u
Wavelength/ nm
y = -0.04374 + 289022.53473*x, R2=0.9955
Absorbance at 421 nm/ a.u
Concentration/ mol.L-1

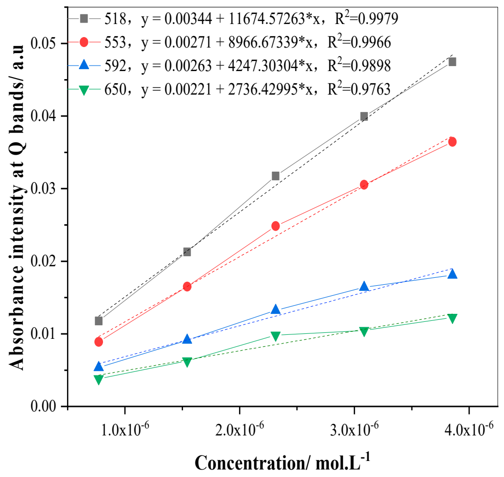
518， y = 0.00344 + 11674.57263*x， R2=0.9979
553， y = 0.00271 + 8966.67339*x， R2=0.9966
592， y = 0.00263 + 4247.30304*x， R2=0.9898
650， y = 0.00221 + 2736.42995*x， R2=0.9763
Absorbance intensity at Q bands/ a.u
Concentration/ mol.L-1

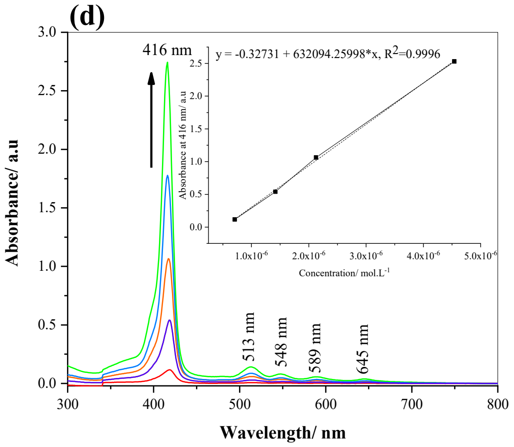
(d)
416 nm
y = -0.32731 + 632094.25998*x, R2=0.9996
Absorbance at 416 nm/ a.u
Concentration/ mol.L-1
513 nm
548 nm
589 nm
645 nm
Absorbance/ a.u
Wavelength/ nm

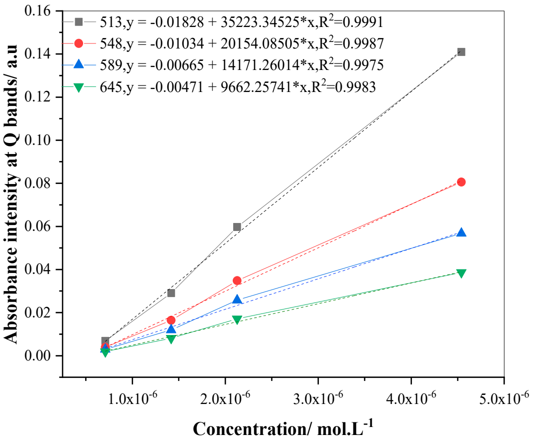
513,y = -0.01828 + 35223.34525*x,R2=0.9991
548,y = -0.01034 + 20154.08505*x,R2=0.9987
589,y = -0.00665 + 14171.26014*x,R2=0.9975
645,y = -0.00471 + 9662.25741*x,R2=0.9983
Absorbance intensity at Q bands/ a.u
Concentration/ mol.L-1

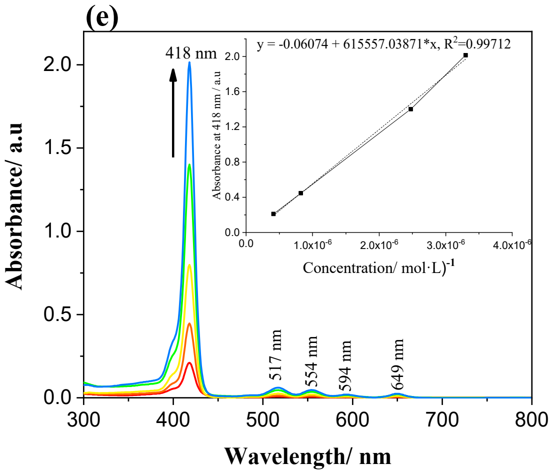
(e)
418 nm
y = -0.06074 + 615557.03871*x, R2=0.99712
Absorbance at 418 nm / a.u
Concentration/ mol·L)-1
517 nm
554 nm
594 nm
649 nm
Absorbance/ a.u
Wavelength/ nm

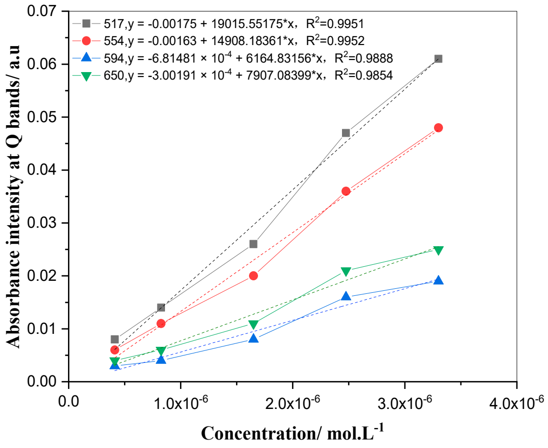
517,y = -0.00175 + 19015.55175*x，R2=0.9951
554,y = -0.00163 + 14908.18361*x，R2=0.9952
594,y = -6.81481 × 10-4 + 6164.83156*x，R2=0.9888
650,y = -3.00191 × 10-4 + 7907.08399*x，R2=0.9854
Absorbance intensity at Q bands/ a.u
Concentration/ mol.L-1

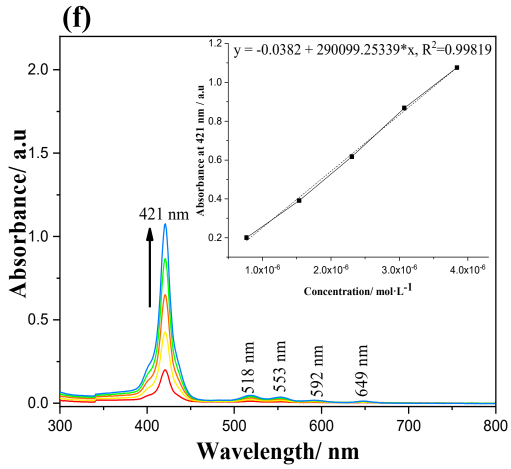
(f)
y = -0.0382 + 290099.25339*x, R2=0.99819
Absorbance at 421 nm / a.u
Concentration/ mol·L-1
421 nm
518 nm
553 nm
592 nm
649 nm
Absorbance/ a.u
Wavelength/ nm

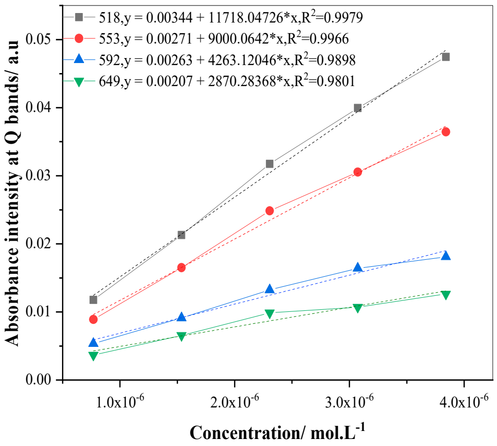
518,y = 0.00344 + 11718.04726*x,R2=0.9979
553,y = 0.00271 + 9000.0642*x,R2=0.9966
592,y = 0.00263 + 4263.12046*x,R2=0.9898
649,y = 0.00207 + 2870.28368*x,R2=0.9801
Absorbance intensity at Q bands/ a.u
Concentration/ mol.L-1

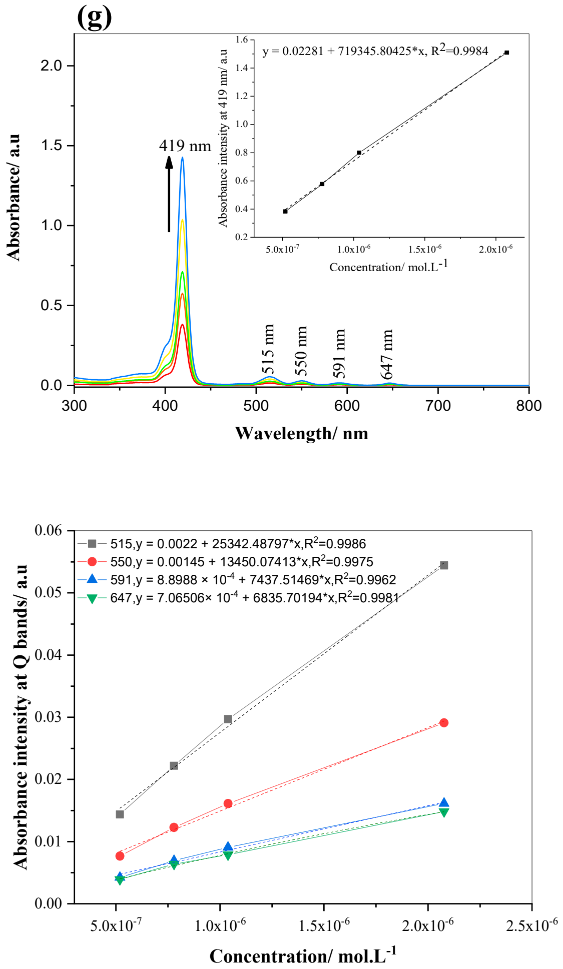

Figure S1. Absorption spectrum of porphyrin solutions with concentration range 7.65 × 10^{-7} ~ 6.12 × 10^{-6} mol·L^{-1} (**a**) TPP in CH_2Cl_2; (**b**) TAPP in THF; (**c**) TBPP in CH_2Cl_2; (**d**) TCPP in CH_3OH; (**e**) THPP in CH_3OH; (**f**) TMPP in CH_2Cl_2; (**g**) TSPP in DMF and the corresponding linear calibrations to calculation of molar absorption coefficients at each band (equals to the slope of each linear equation.
